# Citrullination-acetylation interplay guides E2F-1 activity during the inflammatory response

**DOI:** 10.1126/sciadv.1501257

**Published:** 2016-02-05

**Authors:** Fatemeh Ghari, Anne-Marie Quirke, Shonagh Munro, Joanna Kawalkowska, Sarah Picaud, Joanna McGouran, Venkataraman Subramanian, Aaron Muth, Richard Williams, Benedikt Kessler, Paul R. Thompson, Panagis Fillipakopoulos, Stefan Knapp, Patrick J. Venables, Nicholas B. La Thangue

**Affiliations:** 1 Department of Oncology, University of Oxford, Oxford OX3 7DQ, UK.; 2 Kennedy Institute, University of Oxford, Oxford OX3 7DQ, UK.; 3 Structural Genomics Consortium, University of Oxford, Oxford OX3 7DQ, UK.; 4 Target Discovery Institute, University of Oxford, Oxford OX3 7DQ, UK.; 5 University of Massachusetts Medical School, 55 Lake Avenue North, Worcester, MA 01655, USA.; 6 Ludwig Institute, University of Oxford, Oxford OX3 7DQ, UK.

**Keywords:** E2F-1, PAD4, BRD4, citrullination, cancer, immune response, inflammation

## Abstract

Peptidyl arginine deiminase 4 (PAD4) is a nuclear enzyme that converts arginine residues to citrulline. Although increasingly implicated in inflammatory disease and cancer, the mechanism of action of PAD4 and its functionally relevant pathways remains unclear. E2F transcription factors are a family of master regulators that coordinate gene expression during cellular proliferation and diverse cell fates. We show that E2F-1 is citrullinated by PAD4 in inflammatory cells. Citrullination of E2F-1 assists its chromatin association, specifically to cytokine genes in granulocyte cells. Mechanistically, citrullination augments binding of the BET (bromodomain and extra-terminal domain) family bromodomain reader BRD4 (bromodomain-containing protein 4) to an acetylated domain in E2F-1, and PAD4 and BRD4 coexist with E2F-1 on cytokine gene promoters. Accordingly, the combined inhibition of PAD4 and BRD4 disrupts the chromatin-bound complex and suppresses cytokine gene expression. In the murine collagen-induced arthritis model, chromatin-bound E2F-1 in inflammatory cells and consequent cytokine expression are diminished upon small-molecule inhibition of PAD4 and BRD4, and the combined treatment is clinically efficacious in preventing disease progression. Our results shed light on a new transcription-based mechanism that mediates the inflammatory effect of PAD4 and establish the interplay between citrullination and acetylation in the control of E2F-1 as a regulatory interface for driving inflammatory gene expression.

## Teaser

PAD4-mediated citrullination of E2F-1 transcription factor and its interplay with acetylation affects inflammatory gene expression.

## INTRODUCTION

Peptidyl arginine deiminases (PADs) are a family of five enzymes that mediate hydrolytic deimination (citrullination) of arginine residues. PAD4 is a nuclear member of the family and has been reported to be highly expressed in granulocytes, various cancer types, and pluripotent stem cells (*1*, *2*). Hypercitrullination of histones is associated with the release of neutrophil extracellular traps (NETs) as part of the innate immune response and opening of chromatin in pluripotent stem cells. More specific transcriptional regulatory roles have also been ascribed to PAD4, in relation to both citrullination of specific histone residues and other cellular substrates (*3*, *4*). The growing interest in PAD enzymes as pharmacological targets reflects the increasingly prominent role that they have been reported to play in the context of inflammatory disorders (particularly rheumatoid arthritis) and also cancer.

The E2F family of transcription factors, although historically connected with cell cycle control, is involved with regulating diverse cellular outcomes, including apoptosis, differentiation, metabolism, and inflammation (*5*, *6*). However, the mechanisms that underpin and guide the diverse biological roles of E2F-1 have yet to be identified, although insights from arginine methylation suggest that posttranslational modifications are deterministic. Thus, it is known that methylation in an arginine-rich region influences cell viability or cell death, reflective of the type of methylation event that occurs; PRMT1 (protein arginine methyltransferase 1)–mediated methylation augments apoptosis, contrasting with PRMT5-mediated methylation, which renders E2F-1 with growth-promoting activity (*7*).

Because citrullination occurs on arginine residues (*8*), we reasoned that PAD4 might influence the biological output of E2F-1 if functionally important arginine residues were to be modified. Here, we show that E2F-1 is citrullinated by PAD4 in inflammatory cells, where it augments the chromatin association of E2F-1 to cytokine genes. Significantly, the BET (bromodomain and extra-terminal domain) bromodomain family protein BRD4 (bromodomain-containing protein 4) binds to acetylated lysine residues in E2F-1, and PAD4 regulates the association between E2F-1 and BRD4. Our results show the crucial regulatory interplay between citrullination and acetylation during the inflammatory response and its role in dictating the biological outcome of E2F activity.

## RESULTS

**Fig. 1:**
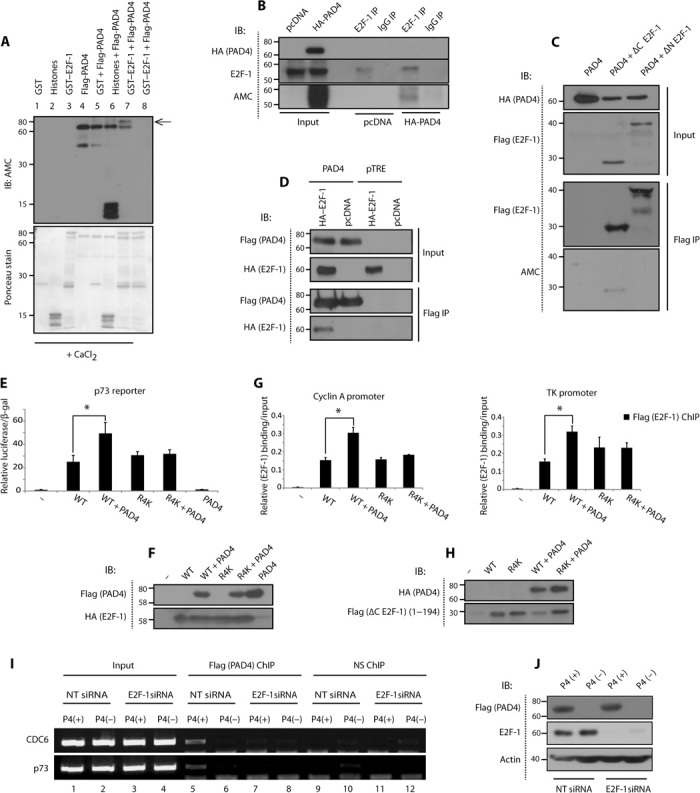
PAD4-mediated citrullination of E2F-1 augments its transcriptional activity. (**A**) In vitro citrullination of GST (glutathione *S*-transferase)–E2F-1by Flag-PAD4, where recombinant GST–E2F-1 (1 μg) was incubated with Flag-PAD4 (1 μg). Lanes 1 to 7 were in the presence of CaCl_2_, and lane 8 in the absence of CaCl_2_. The arrow points to citrullinated GST-E2F-1. AMC, anti-modified citrulline (Millipore); IB, immunoblot. (**B**) Citrullination of E2F-1 in U2OS cells, transfected with HA (hemagglutinin)–PAD4 (2 μg). The lysate was incubated with 4 mM CaCl_2_ and 2 mM dithiothreitol (DTT). E2F-1 (C20) and rabbit immunoglobulin G (IgG) antibodies were used for immunoprecipitation (IP). (**C**) Citrullunation of N-terminal domain of E2F-1 in U2OS cells, transfected with Flag–E2F-1 (1 μg) and HA-PAD4 (2 μg) and treated with A23187 (5 μM, 30 min). Flag-agarose beads were used for immunoprecipitation. ΔC E2F-1: 1 to 194, ΔN E2F-1: 194 to 437. (**D**) Interaction between HA–E2F-1 and Flag-PAD4 in Flag-PAD4.pTRE and pTRE empty vector cell lines transfected with pcDNA or HA–E2F-1 (1 μg). Flag-agarose beads were used for immunoprecipitation. (**E**) Relative luciferase reporter comparing HA–E2F-1 wild-type (WT) and R4K activity in U2OS cells transfected with HA–E2F-1 WT or R4K mutant (R109K/R111K/R113K/R127K) (100 ng), Flag-PAD4 (300 ng), p73 luciferase reporter (100 ng), and β-gal (150 ng) plasmid. The luciferase reporter signal was normalized to β-gal reading. (**F**) Accompanying immunoblot ± SD. *P < 0.05. (**G**) Quantitative polymerase chain reaction (qPCR) analysis comparing Flag ΔC E2F-1 WT and R4K relative promoter occupancy in U2OS cells transfected with Flag ΔC E2F-1 WT or R4K mutant (R^109^/R^111^/R^113^/R^127^) (1 μg) and HA-PAD4 (2 μg). Flag antibody was used for ChIP. (**H**) Accompanying immunoblot ± SD. *P < 0.05. (**I**) ChIP analysis in doxycycline-inducible Flag-PAD4.pTRE cells {treated with or without doxycycline (1 μg/ml) [P4(+) and P4(−), respectively]}, measuring PAD4 promoter occupancy in nontargeting (NT) versus E2F-1 siRNA (30 nM)–transfected cells, and presented as visualized on ethidium bromide–stained gel. NS, nonspecific. (**J**) Accompanying immunoblot.

Successful PAD4-mediated citrullination of recombinant and endogenous E2F-1 was demonstrated in vitro, both biochemically (Fig. 1A, lane 7) and in U2OS cells (Fig. 1B). The comparison of N-terminal (1 to 194) and C-terminal (194 to 437) derivatives (fig. S1A) indicated that the N-terminal region of E2F-1 is more susceptible to citrullination than the C-terminal region (Fig. 1C). Arginine (R) residues 109 and 127 were identified by mass spectrometry as the predominant sites of citrullination (fig. S1, B and C), although site-directed mutagenesis implied that R^111^/R^113^ could also be involved (fig. S1D). Accordingly, E2F-1 and PAD4 were shown to interact in cells (Fig. 1D), principally through the N-terminal region of E2F-1 (fig. S2A). In a gene reporter assay, using the promoter region taken from established E2F target genes (including *TP73* and *CDC6*), exogenous PAD4 increased E2F-1 activity (fig. S2B), whereas PAD4 inhibition reduced E2F-1 activity (fig. S2D) using the PAD4-specific inhibitor TDFA (Thr-Asp-F-amidine) (*9*) (but not the inactive analog TDHA). Furthermore, the noncitrullinated R4K E2F-1 mutant derivative (R109K/R111K/R113K/R127K; see fig. S1A) was not affected by PAD4 relative to wild-type E2F-1 (Fig. 1E). Quantitative measurements of E2F-1 binding to its target gene promoters by chromatin immunoprecipitation (ChIP) revealed that PAD4 enhanced the chromatin association of E2F-1 but not the R4K mutant (Fig. 1G and fig. S2F). PAD4 was itself recruited to E2F-1 target genes, in an E2F-1–dependent manner, because E2F-1 depletion using small interfering RNA (siRNA) diminished PAD4 binding (Fig. 1I). We therefore conclude that PAD4 can enhance the activity and DNA binding properties of E2F-1.

**Fig. 2:**
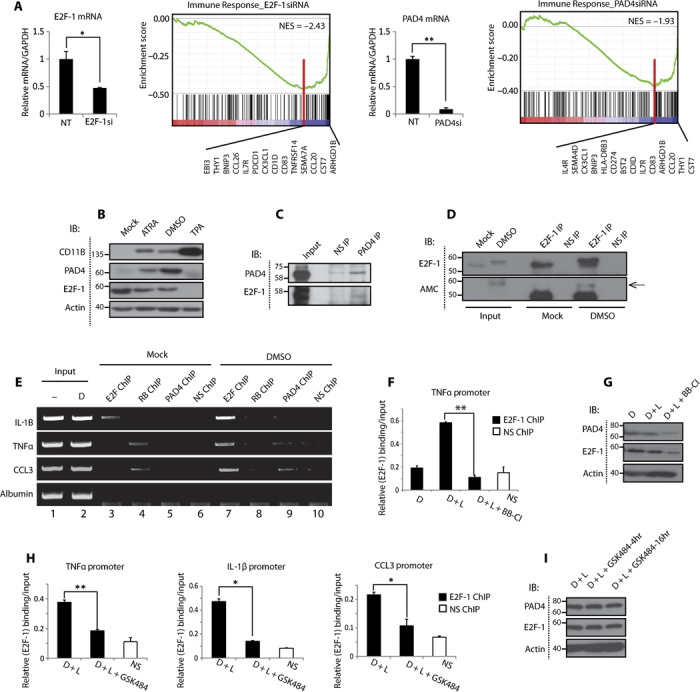
PAD4 regulates the recruitment of E2F-1 to cytokine gene promoters. (**A**) U2OS cells were transfected with nontargeting, E2F-1, or PAD4 siRNA (50 nM). The transcript profile was analyzed using Illumina HumanHT12 v4.0 gene expression array. Transcripts for E2F-1 and PAD4 siRNA (E2F-1si and PAD4si) groups were normalized to nontargeting group and analyzed using GSEA. GSEA revealed down-regulation of immune system (green line) with both E2F-1 and PAD4 siRNAs. The black bars on the far right represent the genes that contribute to the plot. NES, normalized enrichment score. *P* value <0.01 and false discovery rate <0.05, *P < 0.05, **P < 0.01. GAPDH, glyceraldehyde phosphate dehydrogenase. (**B**) Expression of PAD4 in granulocyte-differentiated HL60 cells treated with 1% DMSO, ATRA (1 μM), or TPA (12-*O*-tetradecanoylphorbol 13-acetate) (10 ng/ml) for 48 hours. (**C**) Interaction between E2F-1 and PAD4 in DMSO-differentiated HL60 cells treated with 1% DMSO for 48 hours and immunoprecipitated using PAD4 or rabbit IgG antibodies. (**D**) Citrullination of E2F-1 in DMSO-differentiated HL60 cells. Lysates were incubated with CaCl_2_ (4 mM) and DTT (2 mM) and immunoprecipitated using E2F-1 (C20) or rabbit IgG antibodies. (**E**) ChIP analysis in undifferentiated (-) or DMSO-differentiated (D) HL60 cells. E2F-1 (C20), pRb (IF8), PAD4, and rabbit/mouse IgG antibodies were used for ChIP and presented as visualized on ethidium bromide–stained gel. (**F** to **I**) qPCR analysis in DMSO-differentiated HL60 cells measuring E2F-1 binding to inflammatory gene promoters, treated with LPS (lipopolysaccharide; L) (100 ng/ml, 3 hours) and the pan-PAD inhibitor BB-Cl-amidine (10 μM, 6 hours) (F) or the selective PAD4 inhibitor GSK484 (10 μM, 16 hours) (H), with accompanying immunoblots ± SD (G and I). *P < 0.05, **P < 0.01. Relative E2F-1 protein levels decrease twofold upon BB-Cl-amidine treatment (as quantified by ImageJ), whereas relative E2F-1 promoter occupancy on TNFα promoter decreases fivefold upon BB-Cl-amidine treatment.

We surmised that PAD4-dependent citrullination of E2F-1 might be involved in regulating a subgroup of E2F target genes, to which end we performed transcript profiling in U2OS cells depleted of E2F-1 and PAD4 (Fig. 2A). Pathway enrichment analysis using Gene Set Enrichment Analysis (GSEA) (*10*) revealed significant down-regulation of immune response upon both E2F-1 and PAD4 depletion (Fig. 2A). Furthermore, many of the co-regulated genes were involved in the inflammatory response when analyzed by PANTHER (Protein Analysis Through Evolutionary Relationships) (*11*) (fig. S3, A and B), and the E2F DNA binding motif was enriched in the promoter region of many of these genes as determined by CENTDIST (*12*) (fig. S3, C and D). Given previous reports for the involvement of E2F-1 (*6*) and PAD4 (*3*) in inflammation, we reasoned that PAD4-mediated citrullination of E2F-1 may regulate inflammatory gene expression.

HL60 cells are myeloid leukemic cells that can differentiate to granulocytes with dimethyl sulfoxide (DMSO) or all-trans-retinoic acid (ATRA) (*13*). Consistent with previous reports, we observed a robust induction of PAD4 expression in HL60 cells differentiated into granulocytes (*14*) (Fig. 2B and fig. S4A), with both PAD4 and E2F-1 demonstrating nuclear localization (fig. S4B). In granulocyte-differentiated HL60 cells, we observed an interaction between endogenous E2F-1 and PAD4 (Fig. 2C and fig. S4C), and E2F-1 exhibited increased levels of citrullination (Fig. 2D). We investigated the recruitment of E2F-1 to the candidate inflammatory genes TNFα (tumor necrosis factor–α), CCL3 [chemokine (C-C motif) ligand-3], and IL-1β (interleukin-1β) because they have previously been shown to be up-regulated in differentiated HL60 cells (*15*) and are known E2F-1 target genes (*6*). We observed strong chromatin binding of E2F-1 to the promoters of these cytokine genes in differentiated cells (Fig. 2E, lane 3 versus lane 7), but a decrease in promoter occupancy of pRB (retinoblastoma protein) (Fig. 2E, lane 4 versus lane 8), which negatively regulates E2F-1–dependent transcription (*16*). Increasing levels of E2F-1 on the TNFα promoter were also detected in HL60 cells cotreated with DMSO and TNFα (fig. S4D), which coincided with increased PAD4 activity, because citrullination of histone 3 (H3) (*8*) was enhanced under these conditions (fig. S4E). Crucially, treatment of differentiated HL60 cells with either the pan-PAD inhibitor BB-Cl-amidine (*17*) (Fig. 2F) or the PAD4-specific inhibitor GSK484 (*18*) (Fig. 2H) prevented E2F-1 recruitment to several cytokine gene promoters, thus implying that PAD4 is responsible for targeting E2F-1 to inflammatory genes.

**Fig. 3:**
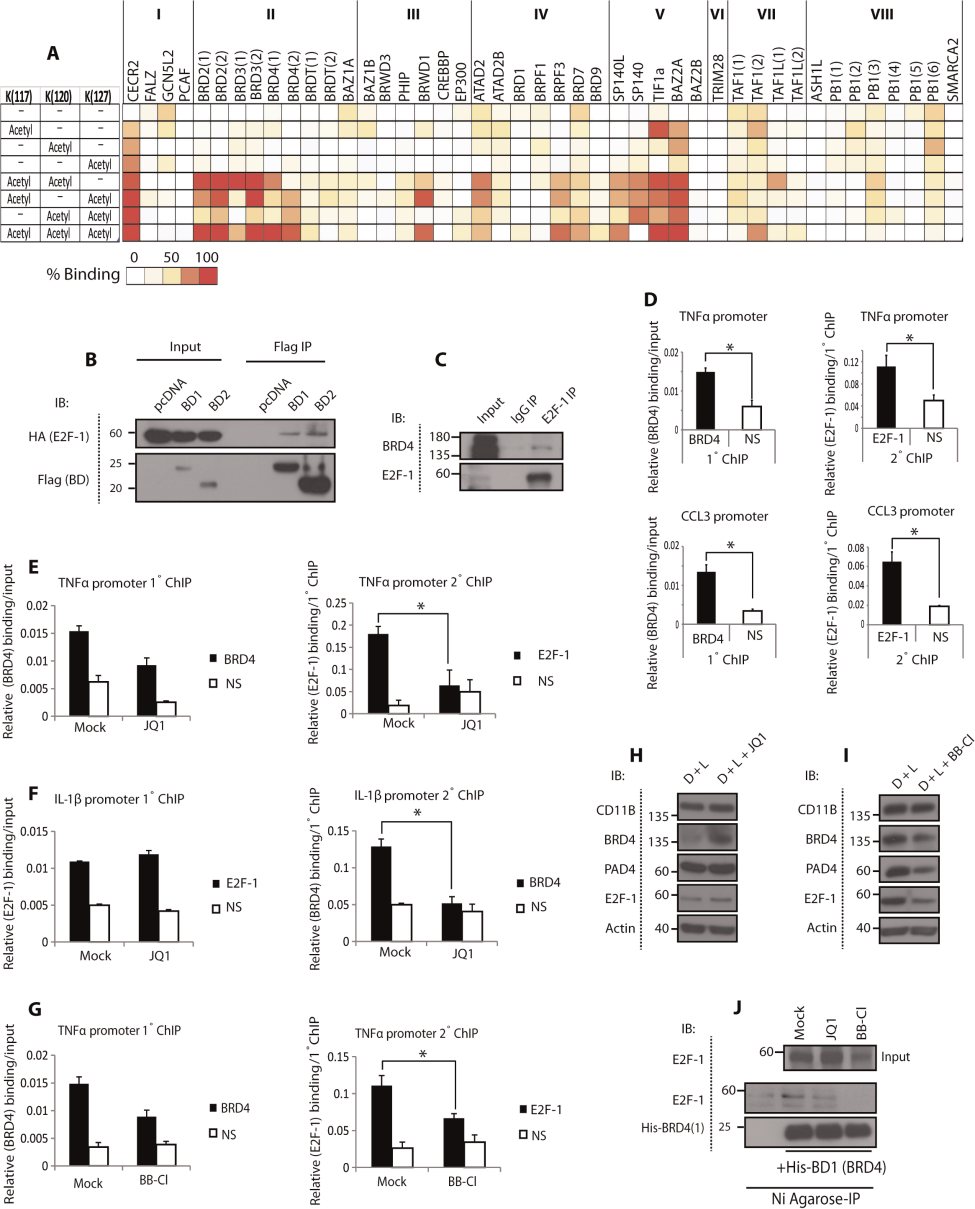
E2F-1 interacts with BRD4 on cytokine gene promoters. (**A**) Heat map of bromodomain binding to acetylated E2F-1 peptides (sequence: 113-RHPGKGVKSPGEKSR-127). The bromodomains are structurally subgrouped into eight families (I to VIII) (*21*), and the multiple domains of a given bromodomain protein are represented by numbers in brackets. (**B**) Interaction between HA–E2F-1 and Flag-BD1/BD2 in human embryonic kidney (HEK) 293T cells transfected with HA–E2F-1 (1 μg) and Flag-BD1 or Flag-BD2 (2 μg) (BD 1 and BD 2 of BRD4, respectively). Flag-agarose beads were used for immunoprecipitation. (**C**) Interaction between E2F-1 and BRD4 in DMSO-differentiated HL60 cells. E2F-1 (C20) or rabbit IgG antibodies were used for immunoprecipitation. (**D** to **I**) qPCR double ChIP analysis of BRD4/E2F-1 interaction in DMSO-differentiated HL60 cells challenged with LPS (100 ng/ml, 3 hours) (D), treated with JQ1 (5 μM, 4 hours) (E and F), or BB-Cl-amidine (2.5 μM, 16 hours) (G), with accompanying immunoblot ± SD (H and I). *P < 0.05, **P < 0.01. (**J**) Interaction between His-BD1 and E2F-1 from U2OS cells treated with JQ1 (5 μM) or BB-Cl-amidine (5 μM) for 16 hours. The cell lysates were incubated with His-tagged BD1 of BRD4 and subject to Ni-agarose immunoprecipitation.

We then investigated the mechanism through which PAD4 augments E2F-1 activity. Because certain bromodomain reader proteins participate in inflammatory gene expression (*19*), we reasoned that recruitment of bromodomains may influence E2F-1 activity during the inflammatory response. We screened a large collection of bromodomains for binding to E2F-1 peptides and identified the BET family, including the two bromodomains of BRD4 [bromodomain 1 (BD1) and BD2], as putative readers of acetylated E2F-1 (Fig. 3A). We focused on BRD4 because it is involved in modulating immune response gene expression (*20*), it is known to bind dual/polyacetylation residues (*21*) (rendering interplay with proximal citrulline marks possible), and its recognition of E2F-1 had not been previously explored. The interaction between E2F-1 and both bromodomains of BRD4 was confirmed in cells (Fig. 3B) and was dependent on the acetylated lysine residues in E2F-1, because E2F-1 K3R, a mutant derivative lacking the sites of lysine acetylation (*22*) (fig. S1A), could not interact with BRD4 (fig. S4F). We found that BRD4 can interact with E2F-1 on cytokine gene promoters (Fig. 3D) in differentiated HL60 cells and endogenous E2F-1 immunoprecipitated BRD4 (Fig. 3C) when using double ChIP. Moreover, the BET inhibitor JQ1 (*23*) was seen to disrupt the E2F-1/BRD4 complex on promoters (Fig. 3, E and F) and in cells (fig. S4G), suggesting that BRD4 interacts with E2F-1 via its bromodomains and drives the expression of inflammatory genes.

**Fig. 4:**
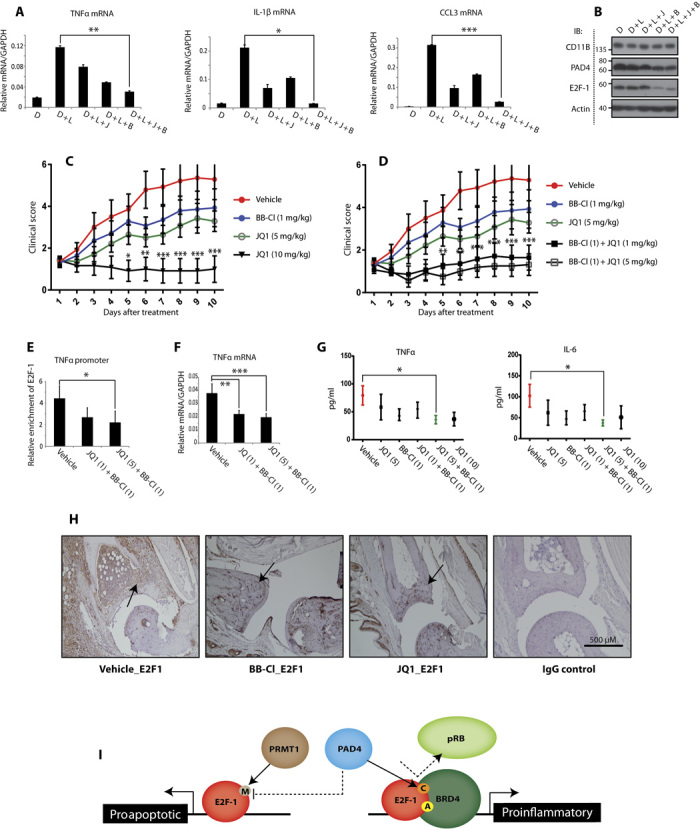
Additive immunosuppressive effects of PAD4 and BRD4 inhibition. (**A** and **B**) Quantitative reverse transcription–PCR (qRT-PCR) analysis of cytokine transcript levels in DMSO-differentiated HL60 cells, treated with LPS (100 ng/ml, 3 hours), JQ1 (100 nM, 16 hours), and BB-Cl-amidine (2.5 μM, 16 hours) where indicated (J, JQ1; B, BB-Cl-amidine) (A), with accompanying immunoblot ± SD (B). *P < 0.05, **P < 0.01, ***P < 0.001. (**C** and **D**) Clinical scores for arthritic paw swelling (*n* = 7 for each treatment) in DBA/1 mice treated with vehicle, BB-Cl-amidine (1 mg/kg), JQ1 (1, 5, or 10 mg/kg), or combinations of, for 10 days from the onset of symptoms. ±SEM. *P < 0.05, **P < 0.01, ***P < 0.001. (**E**) qPCR ChIP analysis of E2F-1 promoter occupancy in murine spleen cells, collected from DBA/1 mice with CIA comparing vehicle and combination treatments. Enrichment of E2F-1 on mouse TNFα gene promoter expressed relative to IgG control. ±SD. *P < 0.05. (**F**) qRT-PCR analysis of TNFα transcript levels in murine spleen cells ± SD. **P < 0.01, ***P < 0.001. (**G**) Secreted levels of TNFα and IL-6 in murine inguinal lymph nodes ± SEM. *P < 0.05. (**H**) E2F-1 and IgG control immunohistochemistry staining of vehicle- and drug-treated paws. The arrows point to cells in the biopsy positively stained for E2F-1. (**I**) Model: PAD4-mediated citrullination targets E2F-1 to inflammatory genes, where E2F-1 interacts with BRD4 to drive inflammatory gene expression.

Having established that PAD4 is important for directing E2F-1 inflammatory gene expression and, further, that BRD4 reads E2F-1 acetylation on inflammatory gene promoters, we surmised that interplay might occur between citrullination and acetylation marks in regulating the inflammatory role of E2F-1. This idea was also suggested from the SPOT array, where an enhanced interaction between E2F-1 peptides and BET bromodomains was apparent when a citrulline flanked an acetyl modification (fig. S4H). To test this idea, we treated differentiated HL60 cells with BB-Cl-amidine and monitored the interaction between E2F-1 and BRD4. Significantly, BB-Cl-amidine treatment reduced the level of the chromatin-bound E2F-1/BRD4 complex as measured by double ChIP (Fig. 3G and fig. S4I), and in U2OS cells treated with BB-Cl-amidine, a reduced interaction between E2F-1 and BD1 bromodomain of BRD4 was evident (Fig. 3J). Thereafter, we measured the expression level of cytokine genes regulated by E2F-1 in cells treated with BB-Cl-amidine and JQ1. Whereas BB-Cl-amidine and JQ1 reduced cytokine expression in cells treated with each compound alone, the combined treatment was able to reduce the expression even further (Fig. 4A).

These results implied a functional interplay between citrullination and lysine acetylation in regulating E2F-1–dependent inflammatory gene expression. We inquired whether similar effects could be recapitulated in vivo in animal models of inflammation and chose to study collagen-induced arthritis (CIA) (*24*). We treated arthritic mice with JQ1, BB-Cl-amidine, or both inhibitors together and monitored any effect on paw swelling. We used a dose of each compound (JQ1, 5 mg/kg; BB-Cl-amidine, 1 mg/kg) that, when administered alone, had a modest effect on disease progression (Fig. 4C). As a control, we administered a high dose of JQ1 (10 mg/kg), which, as expected (*25*), was seen to effectively control paw swelling (Fig. 4C). Crucially, when the two subtherapeutic doses of BB-Cl-amidine and JQ1 (5 or 1 mg/kg) were administered in combination, a marked reduction in clinical symptoms was observed (Fig. 4D).

ChIP analysis on spleen tissue (used as a surrogate tissue source) illustrated that E2F-1 binding to TNFα promoter is diminished in JQ1/BB-Cl-amidine–cotreated mice relative to untreated CIA mice (Fig. 4E), with this coinciding with reduced TNFα transcript levels in the spleen (Fig. 4F). Furthermore, the level of proinflammatory cytokines TNFα and IL-6 measured in the inguinal lymph nodes was reduced with the combination treatment to a level comparable to the treatment with high dose of JQ1 (10 mg/kg) (Fig. 4G). This was consistent with immunohistochemistry pathology examination on paw tissue, which found that the high levels of E2F-1 in the arthritic joints were very much reduced levels in JQ1- or BB-Cl-amidine–treated mice (Fig. 4H). We therefore conclude that the JQ1/BB-Cl-amidine combined treatment reduces the chromatin association of E2F-1 in clinical disease, which reflects reduced cytokine expression and alleviation of clinical symptoms in the CIA mice.

## CONCLUSION

Our results show that PAD4 citrullinates E2F-1 and highlight a novel role for the PAD4–E2F-1 axis in inflammatory gene expression. Previous studies have shown E2F-1 recruitment to the promoters of several inflammatory genes (*6*), and in vivo E2F-1 has been demonstrated to regulate the systemic response to LPSs (*26*). PAD4 has also been reported to regulate the expression of cytokine genes (*3*), and itself becomes activated downstream of various inflammatory stimuli (*27*). Our results provide clarity on a mechanism and pathway through which PAD4 augments the inflammatory response, where E2F-1 recruitment to cytokine genes is regulated by PAD4-mediated citrullination. Furthermore, we identified the BRD4 bromodomain protein as a reader of acetylation marks on E2F-1 during the inflammatory response, which acts together with citrullination to regulate E2F-1 activity. Accordingly, by studying CIA in mice, we highlight the possibility of combining small-molecule inhibitors of PAD4 and BRD4 for treating chronic inflammatory disease, although we recognize that we cannot rule out pathways other than E2F-1 contributing to the observed effects in vivo. Our results bear on the role of regulatory arginines in dictating the biological outcome of E2F-1. Asymmetric methylation on R109 is particularly important for apoptosis (*7*), and this same residue is citrullinated by PAD4. Thus, by virtue of the citrullination event, PAD4 could hinder apoptosis driven by E2F-1 while augmenting its role in the inflammatory response (Fig. 4I). More generally, our study highlights interplay between citrullination and acetylation as a regulatory interface for guiding gene expression.

## MATERIALS AND METHODS

### Cell culture and compound treatments

U2OS and HL60 cell lines were obtained from the American Type Culture Collection. They were maintained in Dulbecco’s modified Eagle’s medium (DMEM) and RPMI-1640 medium (Sigma), respectively, supplemented with 10% (v/v) fetal calf serum (FCS) (Biosera) and 1% (v/v) penicillin-streptomycin (pen-strep) (Gibco). Stable Tet-On cell lines expressing inducible Flag-PAD4 were maintained in DMEM supplemented with 5% (v/v) Tet-negative FCS, G418 (100 μg/ml) (Clontech), 0.3% hygromycin (Clontech), and 1% (v/v) pen-strep. Doxycycline (1 μg/ml) was used to induce PAD4 expression in Flag-PAD4.pTRE cells. HL60 cells were differentiated into granulocyte-like cells upon treatment with 1% DMSO (tissue culture grade, Sigma) or 1 μM ATRA for 48 to 72 hours. They were differentiated into macrophage/monocyte-like cells upon treatment with 10 nM TPA for 48 to 72 hours. The expression of CD11B cell surface marker was used to confirm differentiation. Given the report that a higher percentage of HL60 cells are responsive to differentiation by DMSO than ATRA (*13*), DMSO treatment was used for this study. Where indicated, cells were treated with LPS (100 ng/ml) or TNFα (10 ng/ml) for 3 hours. TDFA and GSK484 are selective PAD4 inhibitors, BB-Cl-amidine is a pan-PAD inhibitor (particularly of isoforms 2 and 4), and JQ1 is a selective BET inhibitor.

### Antibodies

PAD4 (ab128086 and ab50247, Abcam), HA (MMS-101R, Covance), Flag (F3165, Sigma), AMC antibody kit (17-347, Millipore), E2F-1 (KH95 and C20, Santa Cruz Biotechnology), BRD4 (ab128874, Abcam), β-actin (A2228, Sigma), pRB (IF8, Santa Cruz Biotechnology), CD11B (ab75476, Abcam), mouse and rabbit secondary antibodies (GE Healthcare), and HA or Flag antibody-coupled agarose beads (Sigma) were used.

### RNA isolation and PCR

RNA was extracted from cells using TRIzol reagent (Life Technologies) in accordance with the manufacturer’s guidelines. Reverse transcription was preformed using SuperScript III Reverse Transcriptase (Life Technologies), and the complementary DNA (cDNA) was used as template in PCR [using Paq5000 DNA Polymerase (Agilent Technologies)] or qRT-PCR [using Brilliant III SYBR Master Mix (Agilent Technologies)]. For qRT-PCR analysis, transcript levels were normalized to housekeeping gene GAPDH. The primer sequences used are as follows: E2F-1, AAGCCCTGTCAGAAATCCAG (forward) and AGGCCCTCGACTACCACTT (reverse); GAPDH, ACCTTGCCCACAGCCTTGGC (forward) and ATCATCCCTGCCTCTACTGG (reverse); TNFα (human), CGCCGTCTCCTACCAGACCAAGGTCAAC (forward) and ATGATCCCAAAGTAGACCTGCCCAGACTCG (reverse); TNFα (mouse), TACTGAACTTCGGGGTGATTGGTCC (forward) and CAGCCTTGTCCCTTGAAGAGAACC (reverse); IL-1β, AACCTATCTTCTTCGACACATGGGATAACG (forward) and CAAGGCCACAGGTATTTTGTCATTACTTTC (reverse); CCL3, CCTTGCTGTCCTCCTCTGCACCATGGCTCT (forward) and GGTCGCTGACATATTTCTGGACCCACTCCT (reverse); and PAD4, CATGTTCCACCACTTGAAGG (forward) and TCACCTACCACATCAGGCAT (reverse).

### DNA plasmid transfection

Plasmids of interest were transfected into cells using GeneJuice Transfection Reagent (Invitrogen) in accordance with the manufacturer’s guidelines.

### Small interfering RNA

siRNA of interest was transfected into cells using Oligofectamine Transfection Reagent (Life Technologies) in accordance with the manufacturer’s guidelines. E2F-1 siRNA sequence: AACUCCUCGCAGAUCGUCAUC; PAD4 siRNA sequence: GGUCCUGCUACAAACUGUUTT; nontargeting sequence: Dharmacon NT3 control siRNA

### Immunoblotting

Cells were lysed in modified radioimmunoprecipitation assay lysis buffer {150 mM NaCl, 50 mM tris-HCl (pH 7.5), 1% NP-40, 0.1% deoxycholic acid, 1 mM EDTA, 1 mM NaF, 1 mM Na_3_VO_3_, and protease inhibitor cocktail [leupeptin (0.5 μg/ml ), pepstatin (0.5 μg/ml), and aprotonin (0.5 μg/ml)]} and loaded using SDS loading dye [62.5 mM tris-HCl (pH 6.8), 25% (v/v) glycerol, 2% (w/v) SDS, 5% (v/v) β-mercaptoethanol, and 0.0625% (w/v) bromophenol blue] for gel electrophoresis (SDS–polyacrylamide gel electrophoresis).

### Coimmunoprecipitation

Cell lysates were incubated with the antibody of interest (1 to 2 μg) for 1 hour overnight (O/N) at 4°C with gentle rotation. IgA- or IgG-agarose beads (Sigma) were added to the extracts and incubated at 4°C for 1 hour to allow antibody-bead coupling. The beads were washed several times with IP wash buffer [50 mM tris-HCl (pH 7.4), 150 mM NaCl, 0.1% Igepal CA-630/NP-40, 1 mM EDTA, 1 mM NaF, 1 mM Na_3_VO_3_, and protease inhibitor cocktail], and the protein complexes bound to the antibody-agarose beads were eluted by adding SDS loading dye.

### Luciferase reporter assay

Cells transfected with E2F-1 gene promoter-luciferase construct, E2F-1/PAD4, and β-gal plasmids were lysed with Reporter Lysis Buffer (Promega). Luciferase reporter readings were read and normalized to β-gal assay values {β-gal buffer [0.2 M Na_2_PO_4_ (pH 7.2), 2 mM MgCl_2_, 0.7% β-mercaptoethanol, and 0.44 M ortho-nitrophenyl-β-galactopyranoside]}.

### Chromatin immunoprecipitation

Cells were cross-linked with formaldehyde (1%, 10 min) and lysed with ChIP lysis buffer I [5 mM tris-HCl (pH 8.0), 85 mM KCl, 0.5% Igepal CA-630, and protease inhibitor cocktail], followed by ChIP lysis buffer II [10 mM tris-HCl (pH 7.4), 150 mM NaCl, 1 mM EDTA, 1% Igepal CA-630, 1% deoxycholate (DOC), 0.1% SDS, and protease inhibitor cocktail]. The lysates were sonicated to shear the chromatin to fragment sizes between 200 and 1000 base pairs, precleared using IgG antibodies and protein A and G agarose beads, and incubated with the antibody of interest O/N. The protein complex–bound beads were washed with Buffer I [20 mM tris-HCl (pH 8.0), 150 mM NaCl, 2 mM EDTA, 0.1% SDS, 1% Triton X-100, and protease inhibitor cocktail], Buffer II [20 mM tris-HCl (pH 8.0), 500 mM NaCl, 2 mM EDTA, 0.1% SDS, 1% Triton X-100, and protease inhibitor cocktail], Buffer III [10 mM tris-HCl (pH 8.0), 250 mM LiCl, 1 mM EDTA, 1% Igepal CA-630, 1% DOC, and protease inhibitor cocktail], and TE (tris-EDTA) buffer [10 mM tris-HCl (pH 7.4), 1 mM EDTA, and protease inhibitor cocktail]. The protein-chromatin complexes were eluted from beads using elution buffer (1% SDS and 0.1 M NaHCO_3_) and were incubated with Proteinase K (40 μg/ml) and RNAse A (20 μg/ml) and reverse cross-linked at 65°C O/N. DNA was purified using the Qiagen DNA purification kit. The double ChIP experiments were performed in two sequential steps, where the chromatin immunoprecipitated from the primary ChIP was used as the starting material for the secondary ChIP.

The ChIP primer sequences are as follows: E2F-1, AGGAACCGCCGCCGTTGTTCCCGT (forward) and GCTGCCTGCAAAGTCCCGGCCACT (reverse); albumin, TGGGGTTGACAGAAGAGAAAAGC (forward) and TACATTGACAAGGTCTTGTGGAG (reverse); TNFα (human), CGATGGAGAAGAAACCGAGACAGAAGGTG (forward) and AGTTGCTTCTCTCCCTCTTAGCTGGTCCTC (reverse); TNFα (mouse), CGATGGAGAAGAAACCGAGACAGA (forward) and CTCCTGGCTAGTCCCTTGCTGTCCTC (reverse); IL-1β, GTCATATCAATTTATAGTCCCACGCGTAAT (forward) and CTCACACCCCAGATAAAGAGATAACTTGTT (reverse); CCL3, GTCTGAAACCAGCTCTCCTCTTTATAGGCA (forward) and GGACTGACTAAGAATAGCCTTGGGTTGACA (reverse); CDC6, GGCCTCACAGCGACTCTAAGA (forward) and CTCGGACTCACCACAAGC (reverse); TP73, TGAGCCATGAAGATGTGCGAG (forward) and GCTGCTTATGGTCTGATGCTTATG (reverse); cyclin A, CTGCTCAGTTTCCTTTGGTTTACC (forward) and CAAAGACGCCCAGAGATGCAG (reverse); and TK (thymidine kinase), TCCCGGATTCCTCCCACGAG (forward) and TGCGCCTCCGGGAAGTTCAC (reverse).

### In vitro and in-cell citrullination

Active Flag-PAD4 enzyme was purified from HEK293T cells transfected with Flag-PAD4 plasmids. The lysates were immunoprecipitated using Flag-agarose beads, and the enzyme was released from beads using Flag-peptide elution. In vitro citrullination was set up by incubating 0.1 to 1 μg of Flag-PAD4 with 1 to 2 μg of substrate in 20 μl of buffer composed of 500 mM tris-HCl (pH 7.4), 5 mM DTT, and 10 mM CaCl_2_, at 37°C for 1 hour. GST tag alone was used as the negative control, and calf thymus histones were used as a positive control for PAD4-mediated citrullination. The reaction was stopped by the addition of SDS loading buffer and analyzed by immunoblotting using the AMC kit (Millipore). For in-cell citrullination assays, the cells were treated with 5 μM A23187 for 30 min, or the lysate was treated with 4 mM CaCl_2_ and 2 mM DTT for 30 min.

### Drugs for in vivo injection

Stock solutions of BB-Cl-amidine and JQ1 were dissolved in DMSO (Sigma). Before injection, 5 μl of the stock solutions was diluted in 95 μl of 10% (w/v) hydroxypropyl-β-cyclodextrin (Sigma) [prepared in sterile nuclease free water (Promega)], such that the final volume injected into each mouse was 100 μl and the overall DMSO concentration was 5% (v/v).

### Mass spectrometry

Liquid chromatography–tandem mass spectrometry (LC-MS/MS) was performed as previously described (*28*).

### CIA in DBA/1 mice

Arthritis was induced in male DBA/1 mice as previously described (*24*). Briefly, bovine collagen was purified from articular cartilage and dissolved in 0.1 M acetic acid. Ten- to 12-week-old male DBA/1 mice (Harlan UK) received one subcutaneous 100-μl injection of 200 μg of bovine type II collagen in complete Freund’s adjuvant (Difco, BD Biosciences) at the base of the tail and on the flank. After immunization, the mice were monitored daily for signs of arthritis. Once a mouse showed signs of arthritis, it was recruited to a therapy group and monitored daily. Arthritis severity was scored as follows: 0 = normal, 1 = slight swelling and/or erythema, 2 = pronounced swelling, 3 = ankylosis. All four limbs were scored, giving a maximum possible score of 12 per mouse. Seven mice (*n* = 7) were used for each treatment group. All procedures were performed according to the guidelines of the Animals (Scientific Procedures) Act 1986 and the approval of the UK Home Office (project license PPL7335).

### Cell proliferation ex vivo and cytokine detection

Inguinal lymph node cells were cultured for 48 hours in the presence of anti-CD3ε (0.1 μg/ml) (clone: 145-2C11) (eBioscience) or bovine type II collagen (50 μg/ml). Cytokines were detected in lymph node culture supernatants using the Meso Scale Discovery platform according to the manufacturer’s instructions.

### SPOT array

E2F-1 peptides were synthesized on cellulose membrane as previously described (*21*). Briefly, membranes carrying E2F-1 peptides with modified lysine (acetylation) and arginine (citrullination) residues were blocked with 5% (w/v) bovine serum albumin (BSA) in 0.3% Tween 20 phosphate-buffered saline (PBST) for 8 hours at room temperature (RT) and incubated with 1 μM recombinant His-tagged bromodomain at 4°C O/N. The following day, the membranes were washed with PBST buffer and incubated with horseradish peroxidase–conjugated anti-His antibody (Abcam) in 2.5% BSA in PBST for 1 hour at RT. The membranes were then incubated with Pierce ECL Western Blotting Substrate (Thermo Scientific) for 2 min, and luminescence from the membranes was read and captured using the ImageQuant Las 4000 camera.

## Supplementary Material

http://advances.sciencemag.org/cgi/content/full/2/2/e1501257/DC1

## SUPPLEMENTARY MATERIALS

Supplementary material for this article is available at http://advances.sciencemag.org/cgi/content/full/2/2/e1501257/DC1 Fig. S1. Mapping the sites of citrullination on E2F-1. Fig. S2. PAD4 augments E2F-1 activity and DNA binding affinity. Fig. S3. Transcripts overlapping between E2F-1 siRNA– and PAD4 siRNA–treated groups show enrichment of immune response pathways. Fig. S4. E2F-1 is recruited to inflammatory gene promoters. Reference (*29*)
