# Supplementary material for: Citrullination-acetylation interplay guides E2F-1 activity during the inflammatory response
Source: Sci Adv. 2016 Feb 5;2(2):e1501257. doi: 10.1126/sciadv.1501257 (PMC4788482; doi:10.1126/sciadv.1501257)
Supplement: http://advances.sciencemag.org/cgi/content/full/2/2/e1501257/DC1 [file supp_2_2_e1501257__index.html]

Science Advances | Science Advances

## Supplementary Materials

**This PDF file includes:**

- Fig. S1. Mapping the sites of citrullination on E2F-1.
- Fig. S2. PAD4 augments E2F-1 activity and DNA binding affinity.
- Fig. S3. Transcripts overlapping between E2F-1 siRNA– and PAD4 siRNA–treated groups show enrichment of immune response pathways.
- Fig. S4. E2F-1 is recruited to inflammatory gene promoters.
- Reference (*29*)

Download PDF

**Files in this Data Supplement:**

- Adobe PDF - 1501257\_SM.pdf
