# Supplementary material for: Citrullination-acetylation interplay guides E2F-1 activity during the inflammatory response
Source: Sci Adv. 2016 Feb 5;2(2):e1501257. doi: 10.1126/sciadv.1501257 (PMC4788482; doi:10.1126/sciadv.1501257)

Science Advances

AAAS

advances.sciencemag.org/cgi/content/full/2/2/e1501257/DC1

Supplementary Materials for

Citrullination-acetylation interplay guides E2F-1 activity during the inflammatory response

Fatemeh Ghari, Anne-Marie Quirke, Shonagh Munro, Joanna Kawalkowska, Sarah Picaud, Joanna McGouran, Venkataraman Subramanian, Aaron Muth, Richard Williams, Benedikt Kessler, Paul R. Thompson, Panagis Fillipakopoulos, Stefan Knapp, Patrick J. Venables, Nicholas B. La Thangue

Published 5 February 2016, *Sci. Adv.* **2**, e1501257 (2016)
DOI: 10.1126/sciadv.1501257

The PDF file includes:

Fig. S1. Mapping the sites of citrullination on E2F-1.
Fig. S2. PAD4 augments E2F-1 activity and DNA binding affinity.
Fig. S3. Transcripts overlapping between E2F-1 siRNA– and PAD4 siRNA–treated groups show enrichment of immune response pathways.
Fig. S4. E2F-1 is recruited to inflammatory gene promoters.
Reference (*29*)

Supplementary Materials:

SI Figure 1) Mapping the sites of citrullination on E2F-1

(A) Schematic diagram of E2F-1 mutants used in this study. FL E2F-1: Full length E2F-1 (1-437), ΔC E2F-1: N-terminal domain of E2F-1 (1-194), ΔN E2F-1: C-terminal domain of E2F-1 (194-437), K3R: acetylation defective K117R/K120R/K125R mutant, R4K: citrullination defective R109K/R111K/R113K/R127K mutant

(B) Tandem mass (MS/MS) spectra to show citrullination of E2F-1 arginine residues on R109 and **(C)** R127. Matching b and y fragment ions matching and deiminated / citrullinated arginine (R) residues are indicated in red.

(D) U2OS cells were transfected with Flag-E2F-1 WT, R2K (R109K/R127K) or R4K (R109K/R111K/R113K/R127K) (1 μg) and HA-PAD4 (2 μg) and treated with A23187 (5 μM, 30 min). Millipore AMC kit used to detect citrullination. E2F-1 constructs were truncated (1-194). Three representative examples of E2F-1 citrullination illustrated in the figure (#1, #2, #3)

SI Figure 1) Mapping the sites of citrullination on E2F-1

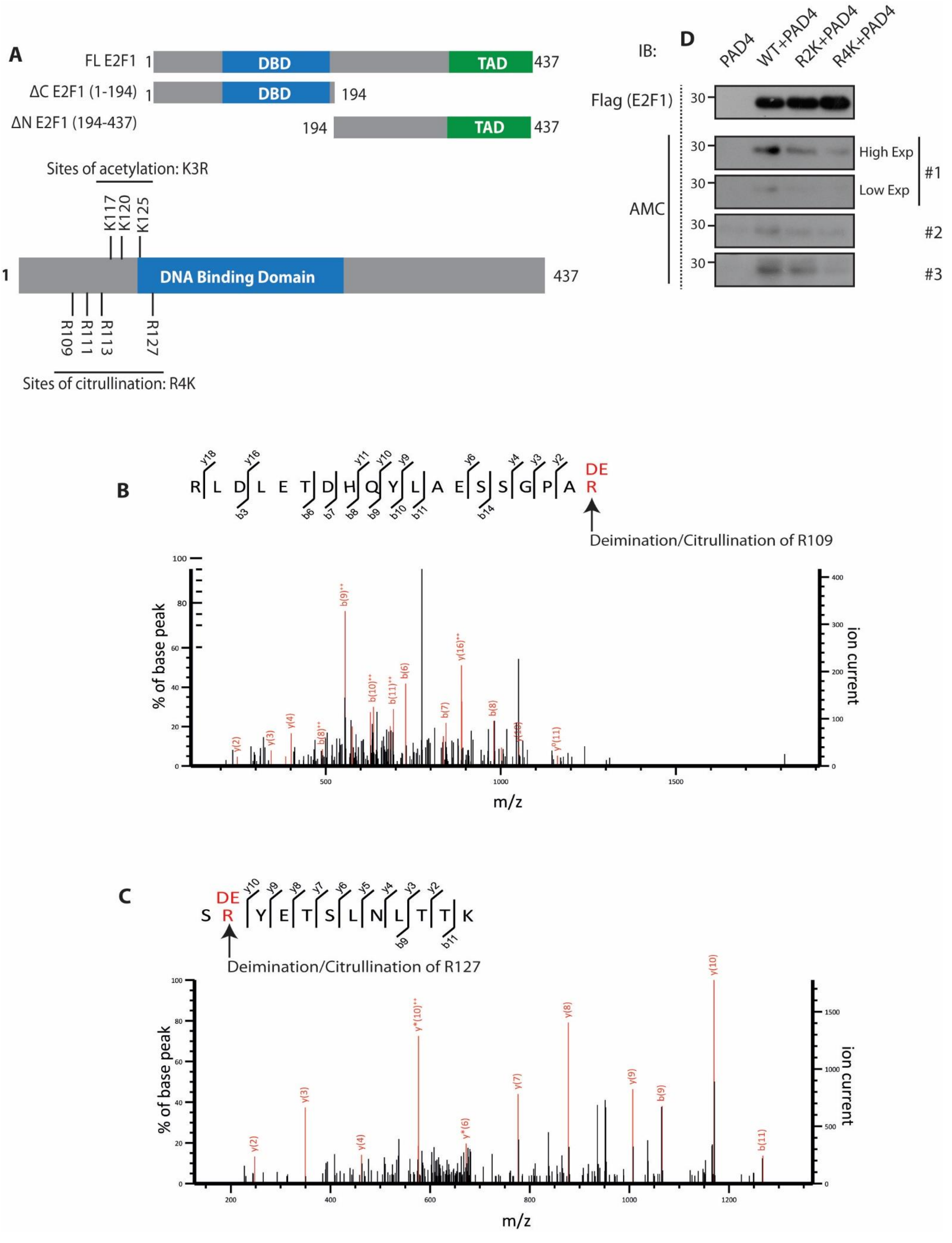

SI Figure 2) PAD4 augments E2F-1 activity and DNA binding affinity

(A) Interaction between truncated Flag-E2F-1 mutant and HA-PAD4 in U2OS cells transfected with Flag-E2F-1 (1 μg) and HA-PAD4 (2 μg). ΔC E2F-1: 1-194, ΔN E2F-1: 194-437 and immunoprecipitated using Flag-agarose beads.

(B) Relative luciferase reporter measuring E2F-1 activity in U2OS cells transfected with HA-E2F-1 (100 ng), Flag-PAD4 (300 ng), CDC6 luciferase reporter construct (100 ng) and β-gal plasmid (150 ng), with **(c)** accompanying immunoblot ± S.D $*p<0.05$

(D) Relative luciferase reporter measuring E2F1 activity in U2OS cells transfected with HA-E2F-1 (100 ng), CDC6 luciferase reporter (100 ng) and β-gal plasmid (150 ng), and treated TDFA or TDHA (5 or10 μM, 16 hr) with **(E)** accompanying immunoblot ± S.D $*p<0.05$

(F) qPCR ChIP analysis measuring relative E2F-1 promoter binding in U2OS cells transfected with Flag-E2F-1 (1 μg) and HA-PAD4 (2 μg), with **(G)** accompanying immunoblot ± S.D $*p<0.05$, $**\ p<0.01$

SI Figure 2) PAD4 augments E2F-1 activity and DNA binding affinity

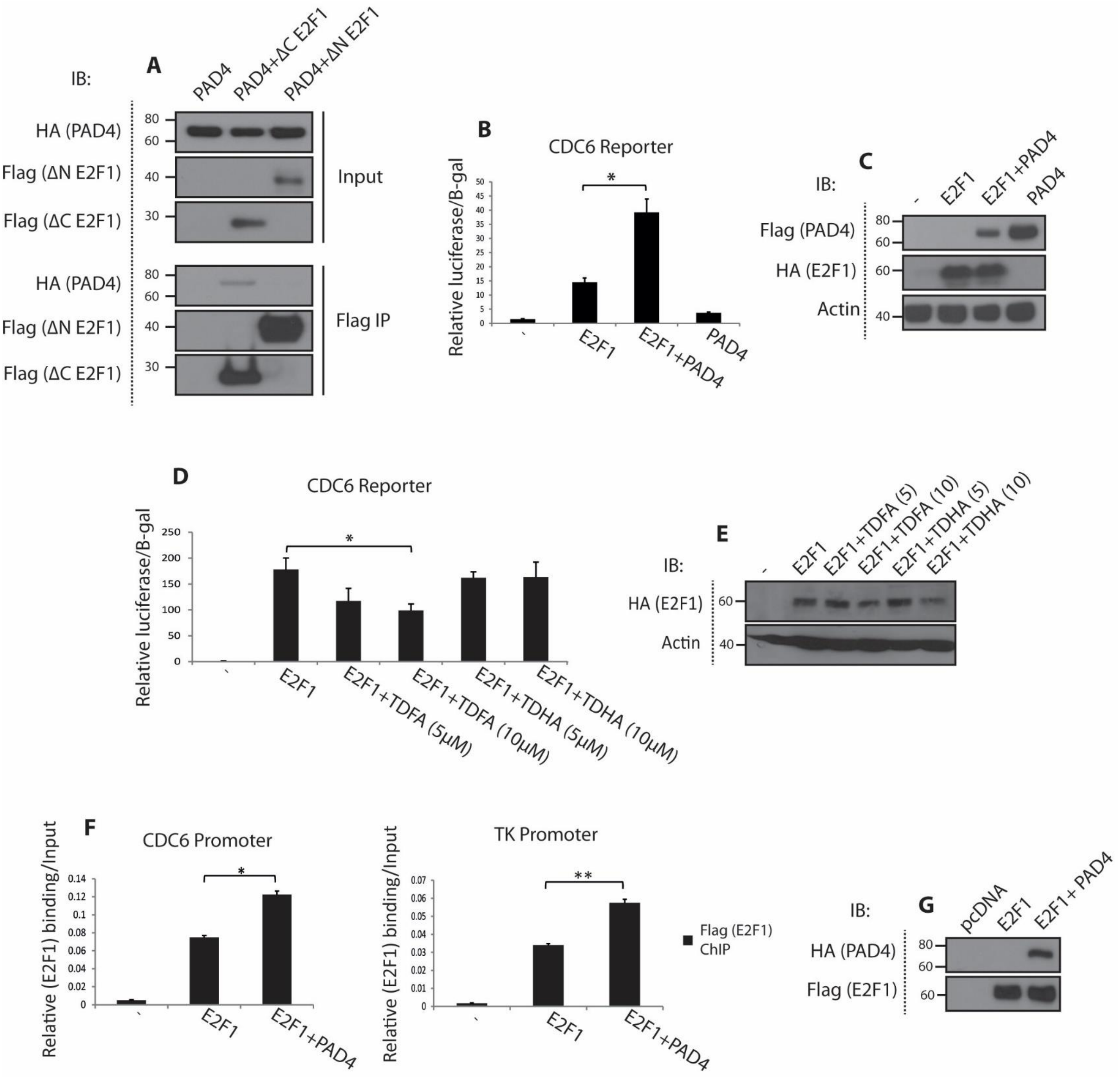

SI Figure 3) Transcripts overlapping between E2F-1 siRNA– and PAD4 siRNA–treated groups show enrichment of immune response pathways

(A) Overlapping set of transcripts between E2F-1 and PAD4 knockdown groups (1406 transcripts) were identified and analysed using the gene set classifier web-based tool PANTHER (PANTHER Overrepresentation Test, version 9.0 Released 2014-01-24) (*11*). Immune system processes was one of the pathways enriched.

(B) List of transcripts which were down regulated when either E2F-1 or PAD4 levels were depleted, and which belong to immune system processes.

(C) Assigned promoter regions of the above genes identified using UCSC Genome Bioinformatics database (Human Feb. 2009 (GRCh37/hg19) Assembly (*29*)).

(D) Promoter regions extracted for these immune response genes were analysed for transcription factor binding site motif using CENTDIST(*12*), from which the E2F transcription factor consensus site (shown as DNA sequence on two strands of DNA) was found to be the third most enriched motif on these promoter regions.

SI Figure 3) Transcripts overlapping between E2F-1 siRNA– and PAD4 siRNA–treated groups show enrichment of immune response pathways

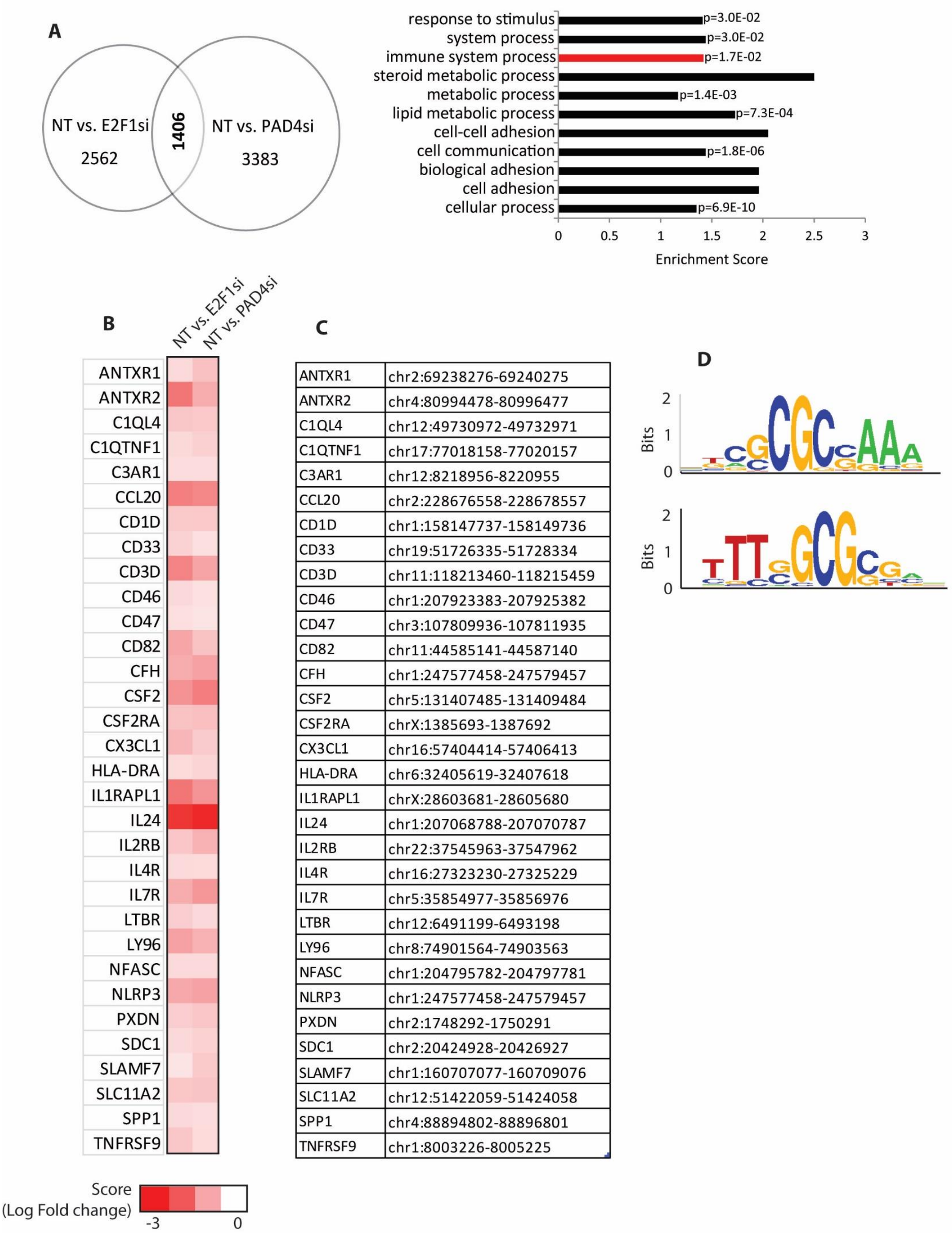

ANTXR1	chr2:69238276-69240275
ANTXR2	chr4:80994478-80996477
C1QL4	chr12:49730972-49732971
C1QTNF1	chr17:77018158-77020157
C3AR1	chr12:8218956-8220955
CCL20	chr2:228676558-228678557
CD1D	chr1:158147737-158149736
CD33	chr19:51726335-51728334
CD3D	chr11:118213460-118215459
CD46	chr1:207923383-207925382
CD47	chr3:107809936-107811935
CD82	chr11:44585141-44587140
CFH	chr1:247577458-247579457
CSF2	chr5:131407485-131409484
CSF2RA	chrX:1385693-1387692
CX3CL1	chr16:57404414-57406413
HLA-DRA	chr6:32405619-32407618
IL1RAPL1	chrX:28603681-28605680
IL24	chr1:207068788-207070787
IL2RB	chr22:37545963-37547962
IL4R	chr16:27323230-27325229
IL7R	chr5:35854977-35856976
LTBR	chr12:6491199-6493198
LY96	chr8:74901564-74903563
NFASC	chr1:204795782-204797781
NLRP3	chr1:247577458-247579457
PXDN	chr2:1748292-1750291
SDC1	chr2:20424928-20426927
SLAMF7	chr1:160707077-160709076
SLC11A2	chr12:51422059-51424058
SPP1	chr4:88894802-88896801
TNFRSF9	chr1:8003226-8005225

SI Figure 4) E2F-1 is recruited to inflammatory gene promoters

(A) HL60 cells were treated with 1% DMSO and harvested at the indicated time points.

(B) DMSO differentiated HL60 cells were fractionated into cytoplasmic and nuclear fractions.

(C) DMSO differentiated HL60 cells were immunoprecipitated using PAD4 or rabbit IgG antibodies.

(D) HL60 cells were treated with DMSO and/or TNFα (10 ng/ml, 3 hr) and $CaCl_2$ (2 mM, 3 hr). E2F-1 (C20) and rabbit IgG antibodies were used for ChIP immunoprecipitation; presented as visualised on ethidium bromide stained gel. **(e)** Immunoblot to show expression levels of PAD4 and citH3

(F) Interaction between HA-E2F-1 and (short isoform) of BRD4 in HEK293T cells transfected with WT or K3R HA-E2F-1 (1 μg). HA-agarose beads were used for immunoprecipitation. K3R: K117R/K120R/K125R

(G) Interaction between HA-E2F-1 and BRD4 in HEK293T cells transfected with HA-E2F-1 (1 μg) and treated with JQ1 (5 μM, 16hr). HA-agarose beads were used for immunoprecipitation.

(H) SPOT array heatmap of the two bromodomains of BRD4 (BD1 and BD2), showing that a combination of an acetyl and a citrulline on E2F-1 peptides improves binding to these bromodomains compared to a single acetyl alone.

(I) DMSO differentiated HL60 cells were treated LPS (100 ng/ml, 3 hr) and BB-Cl-amidine (2.5 μM, 16 hr). BRD4 and rabbit IgG antibodies were used for primary ChIP. The BRD4 primary ChIP was used to perform secondary ChIP with E2F-1 (C20) or rabbit IgG antibodies; presented as visualised on ethidium bromide stained gel. The relative intensities of the specific primary and secondary immunoprecipitations are shown under lanes 3, 5, 7 and 9 respectively.

SI Figure 4) E2F-1 is recruited to inflammatory gene promoters

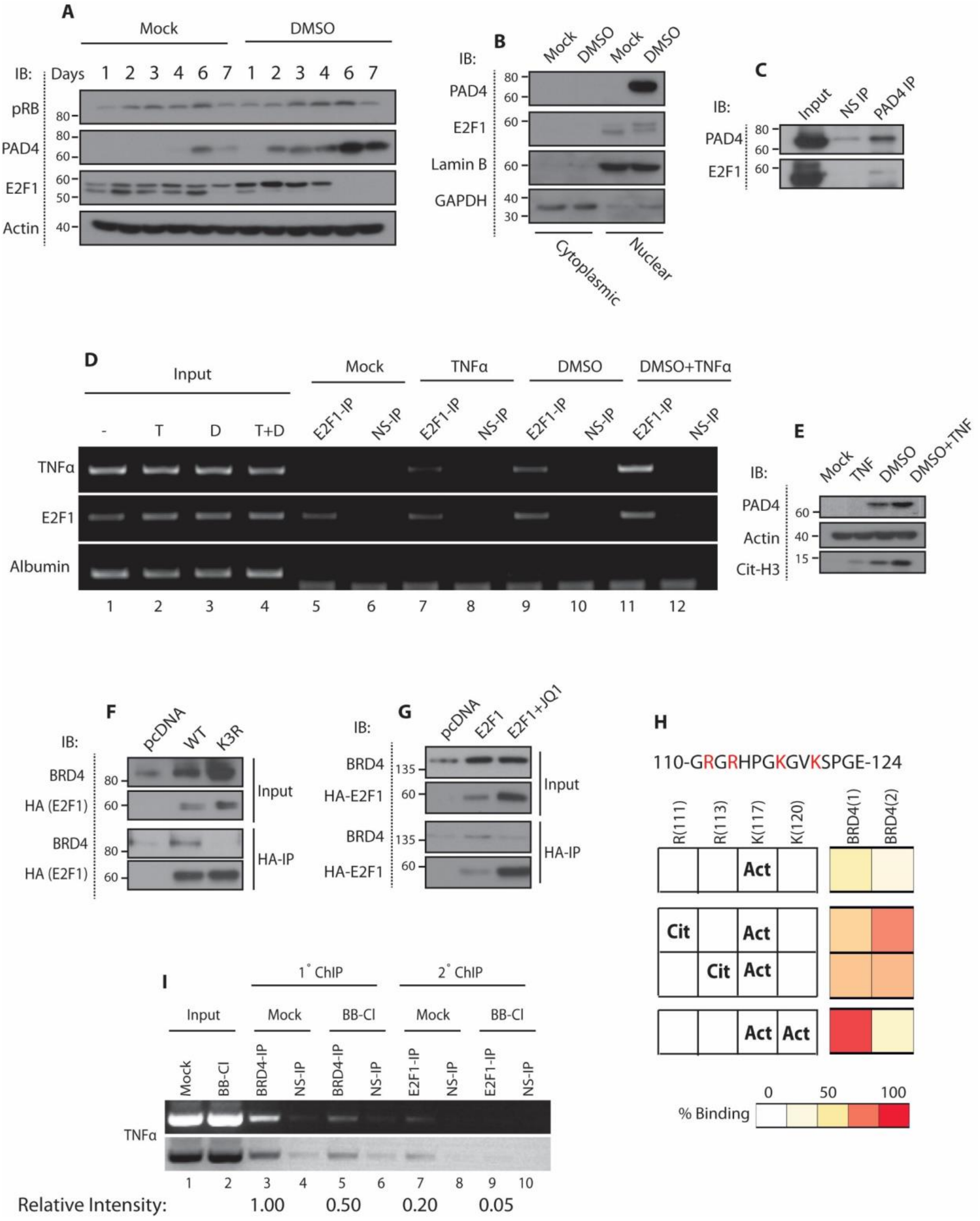

Supplement: http://advances.sciencemag.org/cgi/content/full/2/2/e1501257/DC1 [file 1501257_SM.pdf]
